# *Corynebacterium rouxii,* a recently described member of the *C. diphtheriae* group isolated from three dogs with ulcerative skin lesions

**DOI:** 10.1007/s10482-021-01605-8

**Published:** 2021-06-25

**Authors:** Karen Schlez, Tobias Eisenberg, Jörg Rau, Sabine Dubielzig, Matthias Kornmayer, Georg Wolf, Anja Berger, Alexandra Dangel, Christiane Hoffmann, Christa Ewers, Andreas Sing

**Affiliations:** 1 Hessian State Laboratory, Department of Veterinary Medicine, Hessisches Landeslabor, Schubertstr. 60, 35392 Giessen, Germany; 2 Institute of Hygiene and Infectious Diseases of Animals, Justus Liebig University Giessen, Frankfurter Str. 87-89, 35392 Giessen, Germany; 3 Chemical and Veterinary Analysis Agency Stuttgart, Schaflandstr. 3/2, 70736 Fellbach, Germany; 4 Veterinary Practice for Pets, Wehrenpfad 3, 34560 Fritzlar, Germany; 5 Department for Clinical Veterinary Medicine, Clinic for Small Animal Surgery and Reproduction, Ludwig-Maximilians-University, Veterinärstr. 13, 80539 Munich, Germany; 6 Department of Veterinary Sciences, Institute of Infectious Diseases and Zoonoses, Faculty of Veterinary Medicine, Ludwig-Maximilian-University, Veterinärstr. 13, 80539 Munich, Germany; 7 National Consiliary Laboratory on Diphtheria, Bayerisches Landesamt für Gesundheit und Lebensmittelsicherheit, Veterinärstr. 2, 85764 Oberschleißheim, Germany

**Keywords:** *Corynebacterium diphtheriae*, Dog, Skin lesion, Ulcerative dermatitis, MALDI-TOF MS, *Tox* PCR, NGS

## Abstract

*Corynebacterium* (*C.*) *diphtheriae* is one of the two etiological pathogens for human diphtheria with significant morbidity and mortality. Recently, members of its biovar Belfanti have been described as two novel species, *C. belfantii* and *C. rouxii*. The most important virulence factor and also the premise to cause diphtheria is the isolate’s capacity to encode and express the diphtheria toxin (DT). In contrast to *C. ulcerans,* which represents a potentially zoonotic pathogen, *C. diphtheriae* (incl. the novel deduced species) has almost exclusively been found to comprise a human pathogen. We here report three rare cases of *C. rouxii* isolation from dogs suffering from disseminated poly-bacterial exsudative to purulent dermatitis and a traumatic labial defect, respectively. The isolates were identified as *C. diphtheriae* based on commercial biochemistry and matrix-assisted laser desorption/ionisation–time of flight mass spectrometry (MALDI-TOF MS) analysis. However, recently described specific spectral peaks were highly similar to spectra of *C. rouxii*, which was confirmed by whole genome sequencing. Further investigations of the dog isolates for the presence of DT by *tox* gene qPCR revealed negative results. The findings from this study point out that skin infections in companion animals can be colonized by uncommon and so believed human specific pathogens, thereby resembling the clinical signs of cutaneous diphtheria.

**Supplementary Information:**

The online version contains supplementary material available at 10.1007/s10482-021-01605-8.

## Introduction

*Corynebacterium* (*C.*) *diphtheriae* is the type species of the genus and the etiological microorganism of human diphtheria. Historically and based on DNA homologies, the species *C. diphtheriae*, *C. ulcerans* and *C. pseudotuberculosis* form the *C. diphtheriae* group and have the potential to cause human infections (Pascual et al. 1995; Riegel et al. 1995). However, *C. pseudotuberculosis* is predominantly an animal pathogen, leading to caseous lymphadenitis in small ruminants, horses and camelids (Funke et al. 1997) and *C. ulcerans* can be isolated from a broad number of animal hosts including wild, zoo and companion animals as well as from livestock (Abbott et al. 2020; Berger et al. 2019, 2011; Eisenberg et al. 2015; Foster et al. 2002; Higgs et al. 1967; Hirai-Yuki et al. 2013; Hommez et al. 1999; Marini et al. 2014; Morris et al. 2005; Olson et al. 1988; Schuhegger et al. 2009; Sykes et al. 2010; Venezia et al. 2012). A close contact with both symptomatically and asymptomatically infected companion animals is the preferred route for human transmission. Interestingly, most human diphtheria cases in Western Europe are caused by *C. ulcerans* today (Wagner et al. 2010). In the recent past, systematic changes have led to the description of novel species within the *C. diphtheriae* group. For one of the four biovars of *C. diphtheriae*, namely biovar (bv.) Belfanti, species status has been proposed as *C. belfantii* (Dazas et al. 2018). Atypical strains of the same biovar were the subject of another species description, now designated as *C. rouxii* (Badell et al. 2020). Recently, former *C. ulcerans* strains isolated from game animals (wild boar and roe deer) (Contzen et al. 2011; Eisenberg et al. 2014; Rau et al. 2012), that were provisionally denominated as “wild boar cluster” of *C. ulcerans* (Rau et al. 2019), have now been described as *C. silvaticum* (Dangel et al. 2020). Therefore, the *C. diphtheriae* group comprises now six species.

However, isolations of former *C. diphtheriae* bacteria from animals have rarely been found. Although this organism can be readily cultured in murine macrophage cell lines (Weerasekera et al. 2019), reports of natural infections lastly involved a red fox from Germany caused by *C. diphtheriae* bv. Belfanti (Sing et al. 2016). Furthermore, these authors also give an exhaustive overview of only 11 further similar cases reported during the last century. Besides the single wildlife isolation, infections were otherwise found in companion animals (dogs, cats, horses) and livestock (cattle) with close contact to supposed human shedders.

In contrast to the dermonecrotic exotoxin phospholipase D, a major virulence factor displayed by both *C. ulcerans* and *C. pseudotuberculosis* and involved in caseous lymphadenitis, strains of *C. diphtheriae*, *C. ulcerans* and *C. silvaticum* might carry lysogenic β-corynephages which can harbor a *tox* gene encoded diphtheria toxin (DT), a virulence factor inhibiting protein synthesis (Funke et al. 1997). However, *C. belfantii* and *C. rouxii* strains described so far were all non-toxigenic (Badell et al. 2020) or rarely reported as *tox* gene negative (Dazas et al. 2018). DT contributes to the formation of pseudomembranes in larynx and nasopharynx, which is a hallmark of acute and potentially life-threatening sequelae of diphtheria.

Here we report three cases of *C. rouxii* isolations from dogs in Germany in order to broaden the knowledge on these rare microorganisms and to include them into the differential of skin lesions in dogs.

## Material and methods

### Clinical investigation

#### Case 1

A nearly 6-year old, male, castrated Newfoundlander with a history of disseminated exsudative to purulent dermatitis for few weeks was presented to a local veterinarian. A sample of the external skin of the throat region was submitted for microbiological culture.

#### Case 2

The second dog was a nearly 2-year old female, spayed mixed breed dog with a history of a defect in the right upper lip of supposed shooting trauma resulting in the inability of unimpaired feeding. The dog also displayed purulent nasal discharge and was presented to the university clinic by a society for the prevention of cruelty to animals. The dog was fed by hand with small balls formed of canned dog food. On examination the middle third of the right upper lip, approximately 60% of the width and two thirds of the length of the hard palate were missing. Correspondingly, the nasal cavity was visible over approximately two thirds of its length. The mucosa of the nasal cavity was inflamed; hypertrophied and purulent discharge was present. The complete blood count and chemistry profile were within normal limits. Computed tomography (CT) revealed an osseous defect of the right maxilla and the left mandible. Furthermore, multiple injured teeth, retained tooth roots, various bullet fragments in the soft tissue and enlarged retropharyngeal and mandibular lymph nodes were detected on CT. Accordingly, a gunshot injury with secondary chronic rhinitis was diagnosed. Injured teeth, the retained tooth roots and the teeth of the caudal part of the right maxilla were removed in order to plan soft tissue reconstruction. A sample of the nasal cavity was submitted for microbiological culture and sensitivity testing.

#### Case 3

The third dog was an approximately 2-year-old male castrated mixed breed dog from Romania with a known history of atopic dermatitis and *Malassezia* (*M*.) *pachydermatis* infection. It was presented with otitis externa and a purulent dermatitis accompanied with pruritus, thick and wrinkled skin, lichenification, hyperpigmentation and alopecia on the whole body except the forehead. Samples from the affected skin and the right external ear canal were submitted for microbiological examination.

### Microbiological culture and identification

Swabs were directly streaked on Columbia agar with 5% sheep blood and Gassner’s agar and cultivated using aerobic and microaerophilic atmosphere conditions for 48 h at 37 °C. A brain heart infusion broth was inoculated and incubated in the same fashion to enrich sub-lethally damaged bacteria and plated after 24 h on the above mentioned media. Yeast and mould growth was investigated using a Sabouraud glucose agar with gentamicin and chloramphenicol at 30 °C. All culture media were provided by Oxoid, Wesel, Germany. Isolates were further evaluated using Gram’s staining and matrix assisted laser desorption/ionisation—time of flight mass spectrometry (MALDI-TOF MS; microflex LT Mass Spectrometer, MALDI Biotyper™; Bruker Daltonik, Bremen, Germany) using the direct smear method in sample preparation. Primary identification was done with the commercial MALDI-Biotyper database (MBT 8468; Bruker Daltonik). *Corynebacterium* spp. strains were further identified by biochemical differentiation using the API Coryne system (bioMérieux, Nürtingen, Germany).

### Antimicrobial sensitivity testing

Antimicrobial sensitivity testing (AST) was carried out using broth microdilution testing. Briefly, a commercially available panel layout for pet animals (Micronaut/Bruker according to guidelines of the research group antimicrobial resistance of the German Veterinary Society DVG) was used. In this layout, 14 different antimicrobials were employed ([ranges given in µg ml^−1^]; amoxicillin/clavulanic acid [0.031/0.063–8/16], ampicillin [0.125–8], cephalexin [0.5–16], cefovecin [0.25–4], chloramphenicol [1–16], clindamycin [0.031–2], enrofloxacin [0.016–2], erythromycin [0.125–4], gentamicin [0.063–4], pradofloxacin [0.004–1], oxacillin [0.063–2], penicillin [0.063–4], tetracycline [0.5–8] and trimethoprim/sulfamethoxazole [0.25/4.75–2/38]). Resulting MIC values were interpreted as sensitive, resistant and intermediate resistant by clinical breakpoints according to CLSI M100 29th ed. for broth microdilution testing with special reference to Marosevic et al. (2020).

### Molecular characterization of *Corynebacterium* isolates

The presence of the *tox* gene was investigated by real-time PCR (Schuhegger et al. 2008a). Nearly full-length sequences of the 16S rRNA and *rpoB* gene sequences were deduced from the draft genome sequences and were further used for blast (Yoon et al. 2017). For phylogenetic analysis phylogenetic trees based on nearly full-length 16S rRNA and *rpoB* gene sequences were constructed after alignment with muscle with RAxML (Stamatakis 2014) with the Maximum-Likelihood (ML) method based on General Time Reversible (GTR) model with a discrete Gamma-distribution (+ G) and rapid bootstrap analysis. Both trees are based on 1439–1530 (16S) and 3495–3537 (*rpoB*) nucleotide positions and each 100 replications (bootstrap analysis) (Felsenstein 1985). Isolates were further subjected to *tox* gene qPCR as previously described (Schuhegger et al. 2008b). Whole genome sequencing (WGS) and an average nucleotide identity (ANI) analysis of the sequencing data was done using Illumina MiSeq sequencing, spades assembly and pyani v0.2.7 using ANIb and ANIm as described earlier (Dangel et al. 2020).

## Results

### Bacterial cultures

#### Case 1

After 24 h of incubation, a poly-bacterial growth was noted in the Newfoundlander including *Staphylococcus* (*St*.) *schleiferi*, *Streptococcus* (*S*.) *canis, Escherichia coli* as well as *Acinetobacter baumannii*. However, small coryneform colonies were also noted on Columbia sheep blood agar that were preliminarily identified as *C. diphtheriae* (isolate 191012535 = KL1355).

#### Case 2

The second dog’s microbiological examination revealed growth of *St. aureus*, *St. schleiferi*, *Pseudomonas aeruginosa*, *Neisseria animaloris* and *C. diphtheriae* (isolate 45746 = KL 1306).

#### Case 3

*St. pseudintermedius*, *S. dysgalactiae*, *C. amycolatum*, *M. pachydermatis* and *C. diphtheriae* (isolate 1899/20–5 = KL 1663) were cultured from the skin sample of the third dog. The ear sample revealed growth of *St. pseudintermedius*, *S. dysgalactiae*, *Proteus mirabilis* and *M. pachydermatis*. *C. diphtheriae* could not be detected in the ear canal.

### Phenotypic characterization

**Fig. 1:**
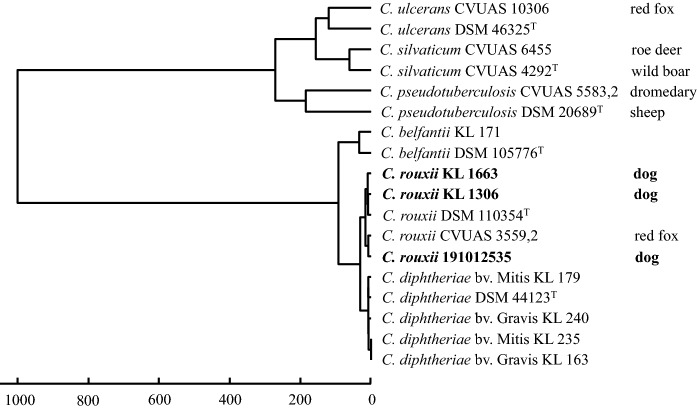
Dendrogram created by cluster analysis of reference main spectra (MSP) obtained by MALDI-TOF mass spectrometry (MALDI Biotyper, Version 3.1, Bruker Daltonik) of three *Corynebacterium* (*C.*) *rouxii* isolates from dogs in comparison to a selection of strains from the *C. diphtheriae* group including spectra of the type strains (^T^) of *C. belfantii*, *C. diphtheriae*, *C. rouxii*, *C. silvaticum*, and *C. ulcerans*. Non-human isolates were indicated with the animal source of isolation. Details of the isolates and reference spectra were given in MALDI-UP (https:maldi-up@ua-bw.bwl.de)

All three corynebacteria isolates were confirmed as Gram-positive coryneform rods and further identified as *C. diphtheriae* by their biochemical patterns (API Coryne code 0010324) showing positive results for glucose and ribose fermentation and for alpha-glucosidase activity and additionally a negative nitrate reaction (Table 1). Using MALDI-TOF MS in combination with the commercial Bruker database (MBT 8468), respective isolates were again identified as *C. diphtheriae* with scores above 2.00, which allows an identification to species level in the previous taxonomy (Rau et al. 2019). The commercial database included 8 reference spectra of *C. diphtheriae* and 280 reference spectra of 79 other *Corynebacterium* species, whereby the currently described species *C. belfantii*, *C. rouxii* and *C. silvaticum* have not yet been included. Consequently, the commercial database was extended by adding custom-made, quality-controlled main spectra projections (MSPs) of the three novel type strains of *C. belfantii, C. rouxii* and *C. silvaticum* to the database (Rau et al. 2016, 2019). Further information regarding the isolates under investigation and an exchange option for the user-made MSPs is provided in the MALDI-UP catalogue at https://maldi-up.ua-bw.bwl.de (Rau et al. 2016). The combined database consisting of the commercial Bruker database and the user-made additions for the type strains of *C. belfantii*, and *C. rouxii* was used for the species decision via MALDI-TOF MS. With this database the MALDI-TOF MS spectra of the isolates from the three dogs were assigned to *C. rouxii* with score values higher than 2.50 for the first hit. Subsequently, a dendrogram was created using MSPs of the three dog isolates from this study as well as from one red fox (Sing et al. 2016) and type strains of the *C. diphtheriae* group (Fig. 1).

**Table 1:** Differentiating phenotypic and genotypic characters within the *C. diphtheriae* group using type strains of the respective taxa. Data for *C. rouxii* were collected from the three canine strains from the present study

Differentiation based on								
Production/ fermentation of	*C. rouxii*	*C. belfantii*	*C. diphtheriae* bv. Mitis	*C. diphtheriae* bv. Gravis	*C. diphtheriae* bv. Intermedius	*C. ulcerans*	*C. silvaticum*	*C. pseudotuberculosis*
Nitrate^£^	−	−	+	+		−	−	−
Pyrazinamidase^£^	−	−					−	
Urease^£^	−	−	−	−		+	+	+
Trehalose^£^	−	−						
Glycogen^£^	−	−	−	+		−	−	−
Maltose^£^	−	+	+	+		+	+	+
16S rRNA gene								
*rpoB* gene								
*tox* gene (Diphtheria toxin)	−	−	+ , − , NTTB	+ , − , NTTB		+ , − , NTTB	NTTB	( +), −
*pld* gene (phospholipase D)						+	+	+
CAMP test (*Rhodococcus hoagii*)	−	−	−	−	−	+	+	+
Inverse CAMP test (*Staphylococcus aureus*)	−	−	−	−	−	+	+	+
Unique MALDI-TOF MS species level	+					+	+	+
Genome size (Mb)	~ 2.4	~ 2.7	~ 2.45	~ 2.45			2.55	

^£^: API Coryne (bioMerieux)

### Antimicrobial susceptibility testing

AST revealed the following minimum inhibitory concentrations (MIC; in µg ml^−1^ in brackets) for respective antimicrobials: amoxicillin/clavulanic acid: (0.125/0.062–0.25/0.125), ampicillin (≤ 0.125–0.25), cephalexin (≤ 0.5–1.0), cefovecin (0.5–1.0), chloramphenicol (≤ 1.0), clindamycin (0.25), enrofloxacin (0.062), erythromycin (≤ 0.125), gentamicin (1.0) and pradofloxacin (0.008–0.016) were found susceptible; resistance was detected for tetracycline (0.125–0.25), oxacillin (1.0–2.0) and trimethoprim/sulfamethoxazole (≤ 0.25/4.75), penicillin was tested intermediate susceptible in case 1 (0.25) and susceptible in cases 2 and 3 (0.125).

### Molecular characterization of *C. rouxii* isolates

Nearly full-length gene sequences were confirmed by 16S rRNA gene sequencing as members of the *C. diphtheriae* group using the curated EzBioCloud database (Yoon et al. 2017). The highest sequence similarity (99.64%) was shared with the type strain of *C. rouxii* FRC0190^T^ (Acc. No. MN535983), followed by *C. diphtheriae* (strain NCTC 11397^ T^ [Acc. No. LN831026]; 99.05%) and *C. belfantii* (strain FRC0043^T^ [Acc. No. OANN01000138]; 98.93%). 16S rRNA and *rpoB* gene sequences were further used for phylogenetic analysis together with other type strains of the *C. diphtheriae* group. The dog isolates from this study clustered in a distinct branch together with *C. rouxii* in both phylogenies (Suppl. Figs. S1, S2). All three isolates were non-toxigenic as shown by a negative *tox* qPCR.

**Fig. 2:**
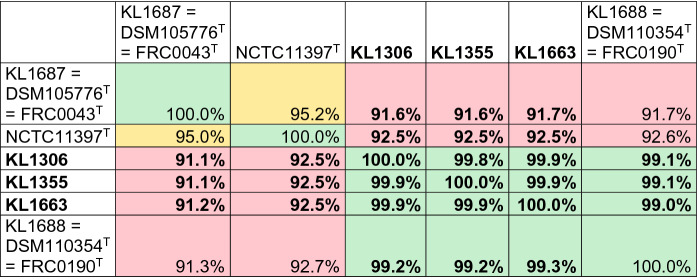
ANIb comparison of dog derived isolates (KL1306, KL1355, KL1663; marked in bold) with type strains NCTC11397^T^ (*C. diphtheriae*), FRC0043^T^ (= DSM105776^T^ = KL1687; *C. belfantii*), FRC0190^T^ (= DSM110354^T^ = KL1688; *C. rouxii).* Boxes with ANI values ≤ 95% are shown in red, with ≥ 94—< 96% in yellow and > 96% in green. ANI comparison with additional data from further *C. belfantii*, *C. rouxii* and additional *Corynebacterium* spp. isolates is given in Suppl. Table S1

The pairwise ANI comparison of the WGS assemblies from the three dog-derived isolates using PyANI (Pritchard 2019) based on MUMmer (ANIm) and blastn + (ANIb) algorithms for reproducibility revealed sequence identities of 91–92% with NCTC11397^T^ (*C. diphtheriae*), 92–93% with FRC0043^T^ (*C. belfantii*), but > 99% with FRC0190^T^ (*C. rouxii*) and are thus well above the species delineation threshold of 95–96% (Goris et al. 2007; Richter and Rossello-Mora 2009) with highest similarity to *C. rouxii* as shown in Fig. 2. The ANI analysis with data from Dazas et al. (2018) and Badell et al. (2020) and type strains of further members of the *C. diphtheriae* group affiliated the three isolates as well with *C. rouxii* (Suppl. Table S1 A and B).

### Treatment regimes

#### Case 1

A topic treatment of the inflamed skin with Pyoderm^®^ shampoo (Virbac Animal Health, Bad Oldesloe, Germany) and Rebohexanid^®^ foam (Alfavet, Neumünster, Germany; twice daily) was administered until AST results were available. The pruritus was treated with oral application of Oclacitinib (Apoquel^®^; Zoetis, Berlin, Germany; 0.4–0.6 mg kg^−1^ q 24 h). The antimicrobial treatment involved amoxicillin/clavulanic acid twice daily orally for one week. Following AST results, treatment was changed to clindamycin twice daily (5.5 mg kg^−1^) orally for another week. The dermatitis resolved completely.

#### Case 2

Two weeks after healing of the mucosa a surgical attempt was performed to close the defect of upper lip and hard palate. Complete closure of the lip and only partial closure of the hard palate was possible due to an injury related anomaly of the *angularis oris* artery and vein. The dog was treated with pain medication and amoxicillin/clavulanic acid (12.5 mg kg^−1^) three-times orally for ten days postoperatively. 20 months postoperatively the dog was able to eat and drink independently, no nasal discharge was present and the cosmetic outcome was good.

#### Case 3

The infection was treated with cephalexin (28.6 mg kg^−1^) twice daily orally and ketoconazole (10 mg kg^−1^) once a day orally over five weeks. Furthermore, the dog was bathed once daily with Maleseb^®^ shampoo (Dechra Veterinary Products A/S, Uldum, Denmark) containing miconazole and chlorhexidine. The pruritus was treated by oral application of Oclacitinib (Apoquel^®^, Zoetis, Berlin, Germany, 0.4–0.6 mg kg^−1^) daily. A hypoallergenic diet (Anallergenic^®^, Royal Canin, Cologne, Germany) was fed for at least eight weeks.

The otitis was treated once a day with Otomax^®^ (MSP Tiergesundheit-Intervet Deutschland GmbH, Unterschleissheim, Germany) containing gentamicin, dexamethason and clotrimazol.

After six weeks the dog was presented with most symptoms in remission but symptoms worsened again later without antibiotic therapy. In two subsequent microbiological examinations (ten and twenty-four weeks after the first microbiological examination) *C. diphtheriae* could no longer be detected.

With respect to zoonotic potential, responsible medical authorities have been informed after receiving initial results of the microbiological culture and sensitivity testing. We are unaware whether testing of owners was conducted. Some of them could be contacted and instructed to wear gloves during handling and to isolate the dog at home. Because clinical signs improved and some dog owners resided far away from the veterinary clinic, follow-up examinations were not conducted in all the cases.

## Discussion

Isolations of bacteria of the *C. diphtheriae* group and of *C. diphtheriae* in particular from animals are rarely reported, but need a thorough evaluation under Public Health considerations. This is even more important in the light of recent taxonomical changes that have not yet been incorporated in the standard microbiological tools used in routine diagnostics (e.g. databases in commercial biochemistry and MALDI-TOF MS). Based on phenotypic as well as molecular data, we provide evidence that the isolates from this study and also an isolate from a red fox from a previous report (Sing et al. 2016) were in fact *C. rouxii* and not *C. diphtheriae* (Figs. 1, 2, Suppl. Figures 1, 2)*.* This underlines the necessity that all putative *C. diphtheriae* isolates in animals need a more in depth approach to be identified to species level. Currently, *C. rouxii* is known from only six isolates that have formerly been assigned to *C. diphtheriae* bv. Belfanti (Badell et al. 2020). These were isolated from human cutaneous or peritoneum infections, one ascitic fluid and also from one dog with purulent orbital cellulitis. Interestingly, another case report describes the finding of *C. diphtheriae* bv. Belfanti, isolated from a cow with dermatitis in Switzerland that should be re-evaluated in the light of recent taxonomical changes (Corboz et al. 1996). It remains to be determined whether the negative *tox* gene is – like in *C. belfantii* – a constant feature in this species, which might directly influence its role as a human diphtheria pathogen. On the other hand, even severe human infections with non-toxigenic strains must not be underestimated with respect to the pathogenic potential (de Santis et al. 2020; Massmann et al. 2020). With four more animal isolates from three dogs from this study and one fox (Sing et al. 2016) and possibly also from two cat isolates from the USA (Hall et al. 2010) it is tempting to speculate and remains to be determined whether *C. rouxii* is also a zoonotic pathogen. Unfortunately, animal keepers were not available for bacterial testing in the here presented cases. Close contact between humans and their pets may foster such transmissions principally in both directions, but – unlike to *C. ulcerans* – the human-to-animal (reverse) zoonosis seems a priori more probable in isolates, formerly known to belong to *C. diphtheriae*. However, *C. rouxii* has already been isolated from a woman suffering from osteomyelitis (Hoefer et al. 2021). In accordance with Sing et al. (2016) the characterization of any *C. diphtheriae* strain isolated from animals – even those sensu lato – is recommended by a reference laboratory as a matter of Public Health concern. For this reason, it is now also important to properly identify isolates of the complete *C. diphtheriae* species complex (*C. diphtheriae* [bv. Gravis, Mitis and Intermedius], *C. belfantii* and *C. rouxii*). The vast minority of the few animal isolates of former *C. diphtheriae* has been subjected or is accessible to profound identification.

With respect to phenotypic traits, Badell et al. (2020) point out that the six *C. rouxii* isolates examined in their study displayed a negative maltose fermentation (utilizing API Coryne) or atypical maltose test results using a Rosco diagnostic method compared to all other *C. diphtheriae* group species. In contrast the three dog isolates from this study displayed a positive maltose test using Api Coryne.

Optimally and like in other species from the *C. diphtheriae* group (including former *C. diphtheriae*, *C. ulcerans*, *C. silvaticum* and *C. pseudotuberculosis*) species differentiation is very well accomplished by MALDI-TOF MS. Presently, the use of commercial databases alone will help to identify the microorganism under study to ‘group-level’, whereas user derived additions to the database have provided great capacity to determine also novel taxa with high accuracy (Dangel et al. 2020; Eisenberg et al. 2014; Rau et al. 2019). Badell et al. 2020 have provided six pairs of *C. rouxii*-specific biomarkers based on MALDI-TOF MS *m/z* signals that facilitate the unequivocal differentiation from other *C. diphtheriae* group species, including *C. belfantii*. These signal patterns are in good agreement with the signals observed for the isolates from the three dogs and for the isolate from a red fox (Sing et al. 2016) (Suppl. Table. S2). User derived reference spectra have been included in the MALDI user platform (www.maldi-up.ua-bw.de) to propagate non-commercial exchange of well suited, quality approved spectra and to improve clinical and veterinary diagnostics with these rarely recognized microorganisms (Rau et al. 2019).

From a clinical perspective, the skin and dermatitis lesions in the dogs of the present study improved under antimicrobial therapy without further complications. Attempts to re-isolate the corynebacteria failed. A number of different bacterial species were isolated in the depicted cases that might also have caused the presented clinical signs alone or in a poly-bacterial combination. Interestingly, also the red fox suffered from severe subacute phlegmonous inflammation of the subcutaneous tissue and a widespread subacute suppurative inflammation of the mammary gland (Sing et al. 2016). However, from a Public Health perspective, antimicrobial sensitivity patterns for the involved *C. rouxiii* isolates were assessed using breakpoints for *C. diphtheriae* in humans. Treatment of *Corynebacterium* spp. can be challenging since Poor et al. (2017) have documented high MIC levels consistent with supposed antimicrobial resistance in sows, especially for macrolides/lincosamide, tetracyclines and quinolones. Contrarily, the dog isolates from this study were found resistant against trimethoprim/sulfamethoxazole, tetracycline and oxacillin only, which represented a favourable starting point for antimicrobial therapy. Based on therapy attempts in *C. ulcerans*, Carfora et al. (2018) recommended topical application of erythromycin for two weeks and the systemic administration of cephalexin for three weeks to treat ulcerative lesions in dogs (Carfora et al. 2018).

## Conclusion

This is the first case series of canine clinical infections with *C. rouxii* isolates with focal to disseminated skin diseases. Generally, *C. diphtheriae*, *C. rouxii* and *C. belfantii* are rarely isolated bacteria in animals. Furthermore, the findings from this study point out that such isolates will be missed by using standard diagnostics, and definitely need further evaluation by expert labs. However, purulent dermatitis in companion animals can be colonized by uncommon and so believed human specific pathogens, thereby resembling the clinical signs of cutaneous diphtheria. Because *C. rouxii* is also a human pathogen, this report broadens the knowledge on this rare microorganism with respect to One Health aspects.

## Supplementary Information

Below is the link to the electronic supplementary material.

Supplementary file1 (XLSX 43 KB)

Supplementary file2 (DOCX 21 KB)

## Data Availability

All data have been made fully available to the public.
