# Supplementary material for: Corynebacterium rouxii, a recently described member of the C. diphtheriae group isolated from three dogs with ulcerative skin lesions
Source: Antonie Van Leeuwenhoek. 2021 Jun 25;114(9):1361–71. doi: 10.1007/s10482-021-01605-8 (PMC8379122; doi:10.1007/s10482-021-01605-8)
Supplement: Supplementary file 2 — Supplementary file2 (DOCX 21 KB) [file 10482_2021_1605_MOESM2_ESM.docx]

**Suppl. Figure S1.** 16S rRNA gene ML tree by RAxML with gtr gamma model and 100 bootstrap rounds, aligned with muscle. Including type strains of *C. diphtheriae* (NCTC11397^T^, biotype gravis), *C. ulcerans* (NCT7910^T^), *C. peudotuberculosis* (ATCC19410^T^), *C. belfantii* (FRC0043^T^and KL1687 = DSM105776^T^), *C. rouxii* (KL1688 = DSM110354^T^), *C. silvaticum* (KL0182^T^) and *C. epidermidicanis* (DSM45586^T^) as outgroup. 16S rRNA gene sequences of *C. diphtheriae* biotypes mitis (strain ISS 3319) and intermedius (strain NCTC 5011) were added. Tree nodes are labelled with isolate name, species name or host species name, where species name has not been assigned before this study, as well as accession number, where available.

**Suppl. Figure S2.** *rpoB* gene ML tree by RAxML with gtr gamma model and 100 bootstrap rounds, aligned with muscle. Including type strains of *C. diphtheriae* (NCTC11397^T^, biotype gravis), *C. ulcerans* (NCT7910^T^), *C. pseudotuberculosis* (ATCC19410^T^), *C. belfantii* (FRC0043^T^and KL1687 = DSM105776^T^), *C. rouxii* (KL1688 = DSM110354^T^), *C. silvaticum* (KL0182^T^) and *C. epidermidicanis* (DSM45586^T^) as outgroup. *rpoB* gene sequences of *C.diphtheriae* biotypes mitis (strain ISS 3319) and intermedius (strain NCTC 5011) were added. Tree nodes are labelled with isolate name, species name or host species name, where species name has not been assigned before this study, as well as accession number, where available.

**Suppl. Table S1.** ANIb (A) and ANIm (B) comparison of dog derived isolates (KL1306, KL1355, KL1663; marked in bold) with additional data from further *C. belfantii*, *C. rouxii* and additional *Corynebacterium* spp. isolates

**Suppl. Table S2.** Position of signals (*m/z*) that are useful for species identification of *C. rouxii* observed by Badell et al., 2020 in comparison with signals observed for corynebacteria isolated from dogs (this study), and a red fox (Sing et al., 2016).

|  | data from Badell et al., 2020 | | | | **dog** | **dog** | **dog** | **red fox** |
| --- | --- | --- | --- | --- | --- | --- | --- | --- |
|  | *C. diphtheriae* (biovar Gravis) | *C. diphtheriae* (biovar Mitis) | *C. belfantii* | *C. rouxii* | 191012535 | KL 1306 | KL 1663 | CVUAS 3559,2 |
|  | n = 3 | n = 10 | n = 8 | n = 6 |  |  |  |  |
| signal /  *m/z* | occurrence:  percentage of absence/  presence of signal | | | | absence (0)/  presence (1) of signal | | | |
| 3255 | *0* | *0* | *0* | *100* | 1 | 1 | 1 | 1 |
| 3263 | *100* | *100* | *75* | *0* | 0 | 0 | 0 | 0 |
| 3610 | *100* | *50* | *100* | *0* | 0 | 0 | 0 | 0 |
| 3640 | *33* | *0* | *0* | *83* | 1 | 1 | 1 | 1 |
| 4732 | *100* | *100* | *100* | *0* | 0 | 0 | 0 | 0 |
| 4748 | *0* | *0* | *0* | *100* | 1 | 1 | 1 | 1 |
| 6512 | *0* | *0* | *0* | *100* | 1 | 1 | 1 | 1 |
| 6529 | *100* | *100* | *100* | *0* | 1 | 1 | 0 | 0 |
| 7222 | *100* | *70* | *100* | *0* | 0 | 0 | 0 | 0 |
| 7281 | *0* | *100* | *0* | *100* | 1 | 1 | 1 | 1 |
| 9463 | *100* | *100* | *88* | *0* | 0 | 0 | 0 | 0 |
| 9495 | *0* | *0* | *0* | *83* | 1 | 1 | 1 | 1 |
|  | | | | | | | | |
| Profile match with data from Badell et al., 2020, weighted by occurrence [%] | | *C. diphtheriae* (bv. Gravis) | | | *11.1* | *11.1* | *2.8* | *2.8* |
|  |  | *C. diphtheriae* (bv. Mitis) | | | *23.3* | *23.3* | *15.0* | *15.0* |
|  |  | *C. belfantii* | | | *11.4* | *11.4* | *3.1* | *3.1* |
|  |  | *C. rouxii* | | | ***88.8*** | ***88.8*** | ***97.2*** | ***97.2*** |
